# Self-Aligned Liquid Crystals on Anisotropic Nano/Microstructured Lanthanum Yttrium Strontium Oxide Layer

**DOI:** 10.3390/ma15196843

**Published:** 2022-10-02

**Authors:** Dong-Wook Lee, Dong-Hyun Kim, Jin-Young Oh, Dae-Hyun Kim, Se-Hoon Choi, Jin-Ah Kim, Hong-Gyu Park, Dae-Shik Seo

**Affiliations:** 1 IT Nano Electronic Device Laboratory, Department of Electrical and Electronic Engineering, Yonsei University, 50 Yonsei-ro, Seodaemun-gu, Seoul 03722, Korea; 2 Department of Smart Electric, Korea Polytechnic, 23 Yeomjeon-ro, 333beon-gil, Nam-gu, Incheon 22121, Korea; 3 Department of Smart Manufacturing Engineering, Changwon National University, 20-1 Changwon Daehak-ro, Uichang-gu, Changwon, Gyeongnam 51140, Korea; 4 Department of Electrical, Electronic, Control Engineering, Changwon National University, 20-1 Changwondaehak-ro, Uichang-gu, Changwon, Gyeongnam 51140, Korea

**Keywords:** lanthanum yttrium strontium oxide, brush-coating, surface morphology, liquid crystal alignment, electro-optical characteristic

## Abstract

We propose an efficient alignment method for liquid crystals (LCs). A brush-coating method handles film deposition and LC alignment treatment simultaneously herein, meaning a reduction in the conventional alignment layer treatment process steps. A lanthanum yttrium strontium oxide (LaYSrO) film prepared by the sol–gel process was used for the alignment layer. Topographical details of the brush-coated LaYSrO films (compared with spin-coated films) were investigated by atomic force microscopy. Spin-coated LaYSrO meant that the film formation alone without orientation treatment represented an isotropic surface. On the other hand, the 270 °C-cured brush-coated LaYSrO showed nano/microstructure with directionality. It indicates that brush-hair sweeping induced shearing stress on the sol state of the LaYSrO, which results in surface anisotropy for LC alignment. The uniform LC alignment state was confirmed by polarized optical microscopy and pretilt analysis. The brush-coated LaYSrO shows fine optical transparency compared to plain and indium-tin-oxide coated glasses, and thermal stability up to 150 °C for LC alignment. Competitive electro-optical performances of the brush-coated LaYSrO were verified in a twisted-nematic LC system compared to those of the conventionally used polyimide layer. Consequently, we expect that the brush-coating process can be an innovative technology for LC alignment.

## 1. Introduction

In modern optics [1,2,3], mechanics [4], and electronics (such as semiconductors [5], light-emitting diodes [6], and smart mirrors [7]), nano/micropatterning has been regarded as a core technology for functional devices. Nano/microstructures can be used for flexible and rollable devices for increased endurance characteristic [4,5], and their periodic and wavy patterns can be used for optical applications, such as organic light-emitting diodes and solar cells [1,2]. In addition, the directional nano/microstructures can induce the surface anisotropy for uniform liquid crystal (LC) alignment [8]. The uniformly aligned LCs can control the light perfectly owing to its unique refractive index anisotropy property and the light controllability is one of the important factors for optical devices such as LC device.

The LCs are placed on the film called alignment layer in the device. Therefore, a film deposition process must first be conducted for the uniform LC alignment. Various film deposition methods have been developed thus far, including chemical vapor deposition [9], sputtering [10], thermal evaporation [11], e-beam evaporation [12], atomic layer deposition [13], and solution coating processing. Among these, solution coating processing is closely related to the orientation and crystallization properties of films, which is an important factor when developing advanced functional devices [14,15]. There are various optical and electronic devices (including electrochemical cell [16], quantum dot LED [17], and light-emitting device [18]) that use the solution coating process. This approach is also appropriate for fabricating high-performance LC devices regardless of inorganic [19,20] or organic [21] alignment layer. Solution-coating methods are mainly categorized into spin-coating [22], bar-coating [23], dip-coating [24], blade-coating [25], and brush-coating processes [26]. In particular, the brush-coating method is appropriate for constructing directional nano/microstructure as it controls the liquid movement by shearing the solution to direct the material.

Conventionally, after the film deposition, the alignment layer treatment process has been conducted for inducing uniform alignment of LCs on that surface. Various alignment layer treatments have been studied, such as ultraviolet photoalignment [27], plasma treatment [28], and the rubbing method [29,30]. Among these, the rubbing method has been used in several industries for a long time. This method induces the surface anisotropy from the microgrooves formed by mechanical contact between the fabric roller and the alignment layer for uniform LC alignment. Although this method produces high-quality performance, it also has several disadvantages such as high cost for large-area processing as well as film deterioration because of contaminant debris generation and cracks [31].

Herein, we adopt the brush-coating process to conduct film deposition and alignment layer treatment at one-step for uniform LC alignment. During the coating process, the brush-hair sweep generated shear stresses on the coated solution, inducing the directional nano/microstructure after the curing process. The formed film could have the physical anisotropy on the surface, which can lead to uniform LC alignment. It indicates that the separate alignment layer treatment process is not required after film deposition. The brush-coating simplified the process steps, and it means the advantages of low cost and high throughput in the process. It is also free of problems caused by the rubbing method. It suggests the potential of the brush-coating process for LC alignment technology. Lanthanum yttrium strontium oxide (LaYSrO) films were prepared for the LC alignment layer by a sol–gel process because of their outstanding electro-optical, nontoxic, and stability properties [32,33,34]. After the brush-coating process, X-ray photoelectron spectroscopy (XPS) analysis was used to confirm the LaYSrO film formation. Atomic force microscopy (AFM) analysis investigated the oriented nano/microstructure formation via brush-coating. Stable, uniform, and homogeneous alignment of the LCs in an antiparallel LC (AP-LC) cell based on the brush-coated LaYSrO layer was examined by polarized optical microscopy (POM) and pretilt angle analysis via the crystal rotation method [35,36]. The thermal budget of the AP-LC cell was evaluated by annealing and POM analysis. The optical transparency of the brush-coated LaYSrO film was observed by ultraviolet, visible, and near-infrared (UV–Vis–NIR) spectroscopy. The crystalline property of the LaYSrO film was measured by X-ray diffraction (XRD). For investigation of the applicability in a twisted-nematic (TN) LC system, the electro-optical (EO) performances were assessed.

## 2. Materials and Methods

### 2.1. LaYSrO Thin-Film Deposition and Alignment Treatment Using Only Brush-Coating Process

**Figure 1:**
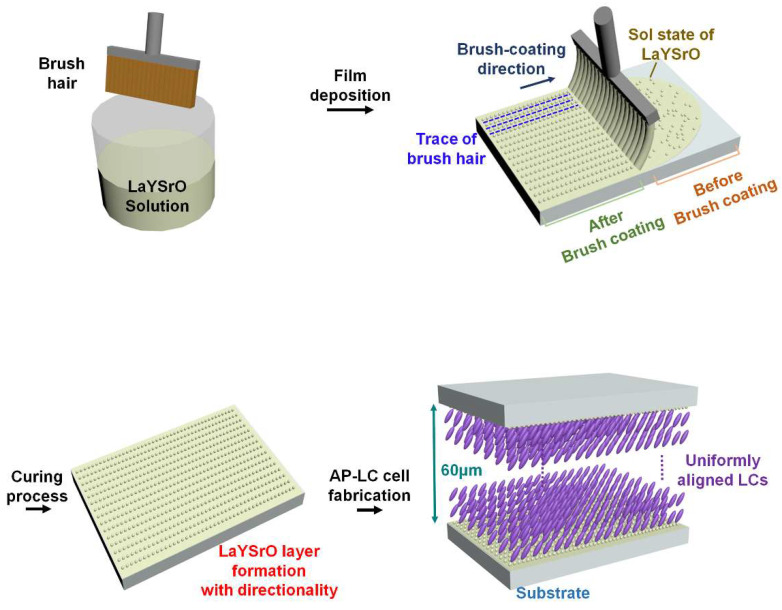
Illustration of the brush-coating process for the LaYSrO solution and the fabricated AP-LC cell. During the coating process, the sol state of the LaYSrO was oriented along the coating direction. Before and after brush-coated regions are presented for intuitive comparison. AP-LC cells with 60 μm cell gap were fabricated based on the directional LaYSrO layer and expected to show uniform alignment of the LCs.

A 0.1 M LaYSrO solution was prepared by the sol–gel process. Lanthanum(III) nitrate hydrate 99.9% trace metals basis [La(NO3)3·xH2O], yttrium(III) nitrate hexahydrate 99.8% trace metals basis [Y(NO3)3∙6H2O], and strontium nitrate 99.995% trace metals basis [Sr(NO3)2] in equal ratios were dissolved in 2-methoxyethanol (2ME). A drop each of acetic acid and mono ethanolamine were then added as stabilizers. Thereafter, the mixture was stirred for 2 h at 420 rpm and 70 °C before aging for at least 1 d. For the substrate, glass (2 × 3 cm) was cleaned using isopropyl alcohol, acetone, and deionized water, with ultrasonication treatment for 10 min before drying with N2 gas. The prepared brush was deeply immersed in the LaYSrO solution for moistening and swept over the glass for simultaneous film formation and alignment treatment [37]. The films were then cured at 70, 170, and 270 °C for 1 h each. The brush-coating process, expected surface modifications, and expected uniform LC alignment on the coated layer are illustrated in Figure 1.

### 2.2. Investigation of the Brush-Coated LaYSrO Film Properties

The chemical compositions of the formed brush-coated LaYSrO films were investigated by XPS (K-alpha, Thermo Scientific, Waltham, MA, USA) using a 12 kV/3 mA power source and monochromatic Al X-ray source (Al Ka line: 1486.6 eV). The surface morphologies of the brush-coated LaYSrO films were then investigated using AFM (NX-10, Park Systems, Suwon, Korea). Spin-coated LaYSrO films were also examined for comparison at the same curing temperatures. The optical transparencies of the brush-coated LaYSrO films were measured by UV–Vis–NIR spectroscopy (JASCO Corporation, V-650) in the wavelength range of 250–850 nm. The film crystallinity was investigated by XRD (DMAX-IIIA, Rigaku, Tokyo, Japan) measurements in the range of 20° to 80° in the 2θ scan mode.

### 2.3. Alignment States and EO Performances of LCs on the Brush-Coated LaYSrO Layer

To evaluate the alignment states of the LCs on the brush-coated LaYSrO layer, antiparallel LC cells were fabricated with uniform 60 μm cell gaps. Positive LCs (IAN-5000XX T14, Δn = 0.111, ne = 1.595, no = 1.484; JNC) were then injected by capillary force. POM (BXP 51, Olympus) observations of the fabricated cells were then performed to clarify the LC alignment states. Pretilt angle analysis using the crystal rotation method (Autronic TBA 107) was implemented to verify the stability and homogeneity of the LCs on the layer. To evaluate the thermal stability of the brush-coated LaYSrO layer in the LC systems, annealing was performed from 90 to 180 °C at equal intervals of 30 °C, with investigation of the LC alignment state in the LC cells by POM analysis. To evaluate the EO capabilities, TN-LC cells were assembled with 5 μm cell gaps. The response time (RT) relevant to the rise and fall times of the LCs as well as voltage–transmittance (V-T) curves related to the threshold voltage were evaluated by an LC device system (LCMS-200).

## 3. Results and Discussions

**Figure 2:**
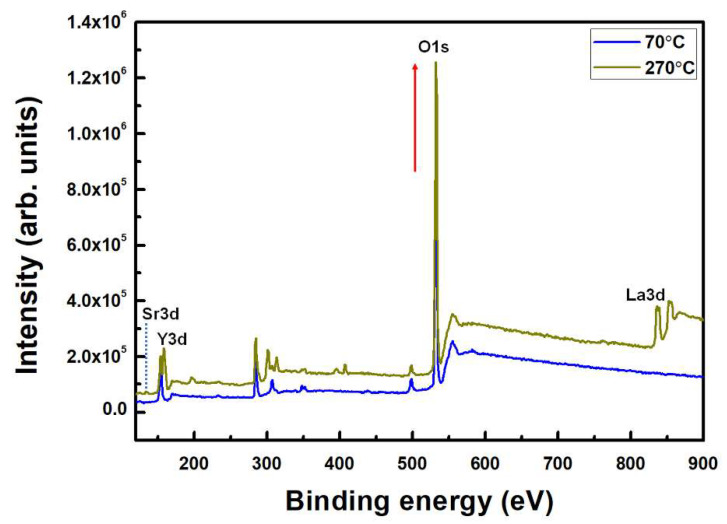
XPS spectra over a wide scan range for the brush-coated LaYSrO films cured at 70 and 270 °C.

As illustrated in Figure 1, the nano/microstructure of the LaYSrO film could achieve directionality on the glass substrate via the brush-coating process. The difference between the regions before (randomly distributed LaYSrO sol state) and after (oriented LaYSrO sol state) the brush-coating progress should be noted. To confirm adequate formation of the LaYSrO film on the brush-coated surface, the stoichiometric characteristics of film cured at 70 and 270 °C were investigated first by XPS, as shown in Figure 2. The Sr 3d, Y 3d, and La 3d peaks were observed for the film cured at 270 °C, where the peaks indicate the main components of the LaYSrO film. However, the film cured at 70 °C showed minute Sr 3d and La 3d peaks, which indicates the unstably oxidized film. In addition, the Y 3d and O 1s peaks had relatively low intensities compared with the film cured at 270 °C. Specifically, the difference in the O 1s peak is very large, which denotes that active thermal oxidation occurred for the film cured at 270 °C. 

**Figure 3:**
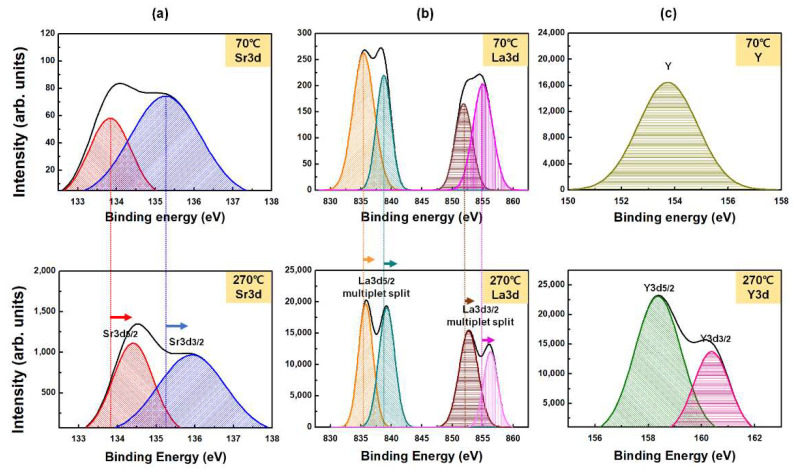
(**a**) Sr 3d, (**b**) La 3d, and (**c**) Y 3d core-level XPS spectra of the brush-coated LaYSrO films cured at 70 and 270 °C.

For specific investigations, the high-resolution Sr 3d, La 3d, and Y 3d XPS data of the LaYSrO films cured at 70 and 270 °C were measured, as shown in Figure 3. The Sr 3d spectra (Figure 3a) were separated into Sr 3d5/2 and Sr 3d3/2 by spin-orbit splitting. The binding energies of Sr 3d5/2 and Sr 3d3/2 were centered at 133.8 eV and 135.2 eV for the film cured at 70 °C and at 134.4 eV and 135.9 eV for the film cured at 270 °C, respectively. The positive peak shift indicates the active oxidized state of the surface of the film cured at 270 °C compared to that cured at 70 °C [34]. The La 3d spectra (Figure 3b) were separated into La 3d5/2 and La 3d3/2 by spin-orbit splitting, and each spin-orbit component was further split into two peaks by multiple splitting. The two peaks belonging to the La 3d5/2 component had binding energies centered at 835.4 eV and 838.7 eV for the film cured at 70 °C and at 835.8 eV and 839.2 eV for the film cured at 270 °C, respectively. The two peaks belonging to the La 3d53/2 component had binding energies centered at 851.8 eV and 855.0 eV when cured at 70 °C and at 852.7 eV and 856.3 eV when cured at 270 °C, respectively. Similar to the Sr 3d spectra, the La 3d peaks were slightly shifted towards the positive side and represent the active thermal oxidized states of the film cured at 270 °C [32]. The Y 3d spectrum (Figure 3c) showed a single peak centered at 153.7 eV for the film cured at 70 °C; this denotes the metal state of yttrium on the film and indicates that there was not enough heat for oxidation during the curing process. On the other hand, in the film cured at 270 °C, the Y 3d spectra were separated into Y 3d5/2 and Y 3d3/2 by spin-orbit splitting, and the binding energies of Y 3d5/2 and Y 3d3/2 were centered at 158.4 eV and 160.4 eV, respectively, indicating adequate formation of the oxide film on the surface [33]. From the chemical composition investigations by XPS, suitable LaYSrO film formation was demonstrated on the glass substrate for the film formed by the brush-coating process and cured at 270 °C.

**Figure 4:**
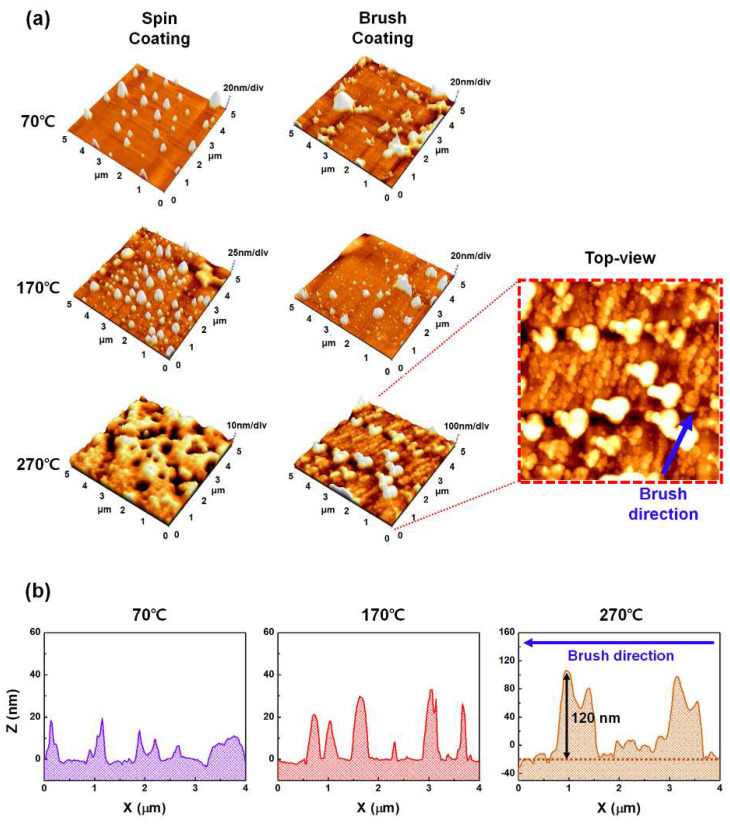
(**a**) Three-dimensional surface morphologies of the spin-coated and brush-coated LaYSrO films observed by AFM. The films were cured at 70, 170, and 270 °C, respectively. Particularly, the brush-coated film cured at 270 °C represents nano/micropatterns with a directionality similar to that of the brush-coating direction (blue arrow shows brush-coating direction). (**b**) Line profiles of the brush-coated LaYSrO films cured at 70, 170, and 270 °C.

The three-dimensional morphological images of the LaYSrO films fabricated by spin-coating and brush-coating at curing temperatures of 70, 170, and 270 °C are presented in Figure 4a. Isotropic surface characteristic was observed for the spin-coated LaYSrO film surfaces, which were not appropriate for uniform LC alignment. Spin-coating uses centrifugal force with high-speed rotation, thereby ensuring that there is no directionality on the surface. Specifically, many particles were observed at relatively low curing temperatures (70 and 170 °C in this case), which were attributed to the residual solvent effect. The spin-coated surface cured at 270 °C was smooth, flat, and isotropic. Therefore, the spin-coating process alone cannot result in uniform alignment of the LC molecules. On the other hand, the brush-coated LaYSrO films cured at relatively low temperatures (70 and 170 °C in this case) represented rough and unstable surfaces with large agglomerates; this attributed to the aggregation of LaYSrO sol between the brush hairs during the coating process as well as the residual solvent effect. There seems to be neither an isotropic surface nor directionality. At 270 °C curing, the brush-coated LaYSrO surface exhibited directionality, as per expectation, and the top-view image is shown for accurate observation. The oriented nano/microstructures are aligned in the same direction as the brush-coating (denoted by blue arrow in the figure). To investigate the morphology and feature size of the surface more concretely, line profile data of the brush-coated films were measured, as shown in Figure 4b. The films cured at 70 and 170 °C exhibited irregular surface morphologies without any periodicity. On the other hand, the surface morphology of the film cured at 270 °C showed repeated increases and decreases in height along the brush-coating direction; this indicated that the recession of brush hairs on the surface resulted in the retracing force of the bulk LaYSrO solution and that the force induced a shearing stress on the sol state of the LaYSrO while the solution was solidified by curing. The formed directional structure corresponded with our expectation shown in Figure 1. This unidirectionally aligned nano/microstructure (such as microgrooves) had a pitch of 2.2 μm and height of 120 nm. This anisotropic structure could cause anisotropy on the surface, and it can ensure uniform alignment of the LC molecules on that surface because of their inherent collective behavioral characteristics with fluidity at the interface [38,39,40,41]. Therefore, the brush-coated LaYSrO films cured at 270 °C are suitable for uniform LC alignment layer.

**Figure 5:**
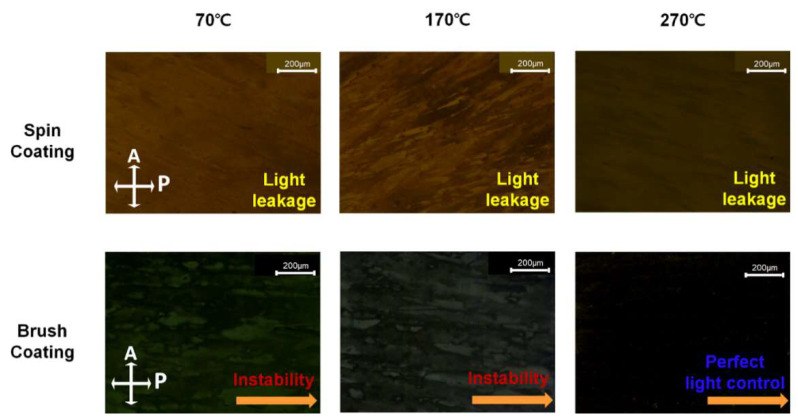
POM results of the AP-LC cells fabricated by spin-coated and brush-coated LaYSrO layers cured at 70, 170, and 270 °C. The analyzer (A) and polarizer (P) are denoted by white arrows. The brush-coating direction is represented by thick orange arrow.

To confirm the alignment state of the LCs on the brush-coated LaYSrO layer, AP-LC cells were fabricated, and POM measurements were obtained, as shown in Figure 5. The spin-coated LaYSrO-layer-based LC cells were also observed for comparison. The spin-coating-based LC cells generally show yellowish images, which indicate the light leakage effect because of the randomly distributed LCs in the cell. This result corresponded with the AFM analysis, which showed isotropic characteristic on the spin-coated film surfaces. As the curing temperature increased, the film became free of residual solvents, and the stability of the film increased while still showing a yellowish image. The brush-coating-based LC cells generally exhibited dark images, but partial light scattering and instability were observed in the layers cured at 70 and 170 °C. This means that relatively low curing temperatures do not provide directionality or allow suitable direction of the LC matrix without anisotropy. The brush-coated LaYSrO layer cured at 270 °C showed distinct dark images, indicating that uniform LC alignment was achieved in a single direction without dislocations on the stable alignment layer. The uniformly aligned LCs could guide the light path passing through the LC cells, and the path-guided light was blocked by the vertically crossed analyzer and polarizer, thereby generating a perfect dark image for the uniformly aligned LC layer. The analyzer and polarizer directions are denoted by the white arrows in the figure. Further, for specific analysis of the uniform LC alignment mechanism, we consider the surface nano/microstructure of the brush-coated LaYSrO film cured at 270 °C. The directionality of the structure was along the brush-coating direction, and this anisotropic surface generated geometric restrictions at the boundary between the interface and LCs. Because of the fluidity of their collective behavioral characteristics, which is derived from the van der Waals forces between the LC molecules and accompanied by elastic distortions controlled by the boundary conditions such as microgrooves, the LC molecules spread their orientation characteristics through the bulk state LC molecules in the cell to achieve uniform alignment. The POM results also correspond with the AFM analysis, and the brush-coated LaYSrO layer cured at 270 °C was demonstrated to have a uniform LC alignment layer.

**Figure 6:**
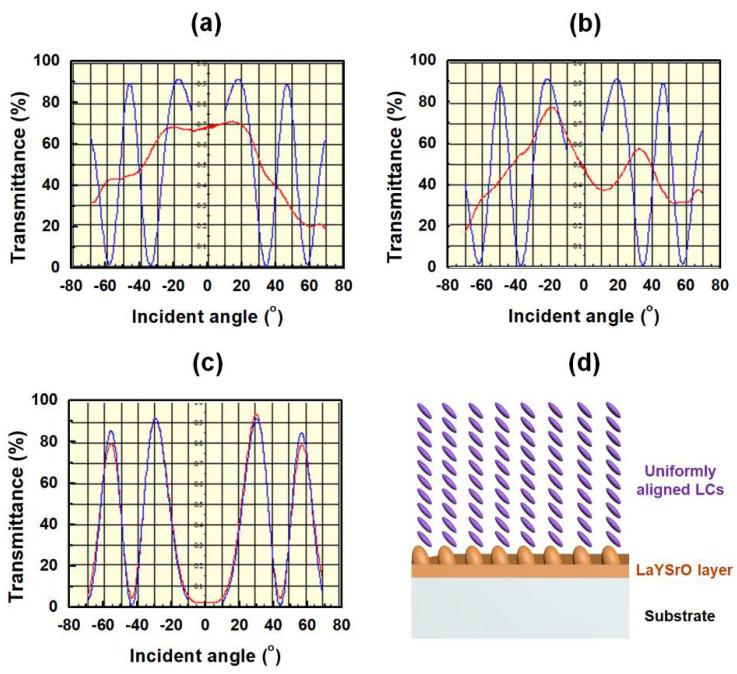
Optical transmittance graphs of the AP-LC cells fabricated from brush-coated LaYSrO films cured at (**a**) 70 °C, (**b**) 170 °C, and (**c**) 270 °C. The graphs are obtained by latitudinal rotation from −70° to 70° using the crystal rotation method. (**d**) Illustration of uniform and homogeneous LC alignment on the oriented nano/microstructures of the constructed LaYSrO alignment layer.

To investigate the pretilt angles of the LCs in the cells fabricated using the brush-coated LaYSrO layer, the optical transmittance curves were measured using the crystal rotation method by rotating the cell latitudinally from −70° to +70°. The result graphs are shown in Figure 6a–c, where the blue lines indicate simulation data and red lines show the experimentally measured data. The LC cells made from the LaYSrO layer cured at 70 and 170 °C (Figure 6a,b) showed irregular and mismatched rates between simulations and experiments. On the other hand, the LC cells made from the layer cured at 270 °C (Figure 6c) showed high accordance rate between the two lines and regular curve of the graph. This showed the stability of the LaYSrO layer, uniform LC alignment on the layer, and guaranteed the reliability of the pretilt angles calculated from the graphs. The calculated pretilt angle was 0.16°, which indicated homogeneous alignment of the LC molecules. The homogeneous (planar) alignment of LC molecules in the anisotropic boundary nano/microgrooves constructed on the surface can be explained by the Berman model [40,41]. From the POM and pretilt angle analyses, it is demonstrated that the brush-coated LaYSrO layer cured at 270 °C can achieve the homogeneous and uniform LC alignment and that the brush-coating process is suitable for LC alignment technology. The illustration of the uniform and homogeneous alignment of the LC molecules along the brush-coated LaYSrO surface directionality is presented in Figure 6d.

**Figure 7:**
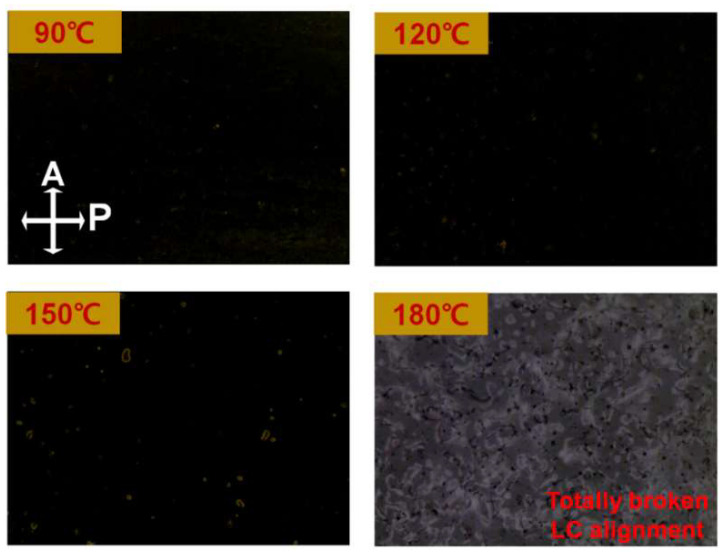
Thermal stability tests of the LCs in the AP-LC cell made from brush-coated LaYSrO layers cured at 270 °C. Annealing was performed from 90 °C to 180 °C in steps of 30 °C for 10 min at each temperature, and the results were measured by POM.

Advanced LC applications for high-resolution displays require high thermal budget because of the numerous switching components. To examine the thermal budget of the LC cells assembled from the brush-coated LaYSrO layer cured at 270 °C, POM measurements and annealing were performed, as shown in Figure 7. Heat was applied from 90 °C to 180 °C in increments of 30 °C for 10 min, followed by gradual cooling to 25 °C. Similar to the clear dark image observed at room temperature, a perfect dark image was obtained up to 120 °C, indicating the stable and uniform LC alignment state. Very few and small defects were observed at 150 °C, which still showed an overall dark image indicating maintenance of orientation of the LC molecules in the cell. However, when the heat was increased to 180 °C, a grayish image was observed, indicating disintegration of the LC alignment and random distribution. From the thermal stability tests, it was demonstrated that the brush-coated LaYSrO layer had an appropriate thermal budget for LC devices compared to the conventionally used rubbed polyimide layer [42].

**Figure 8:**
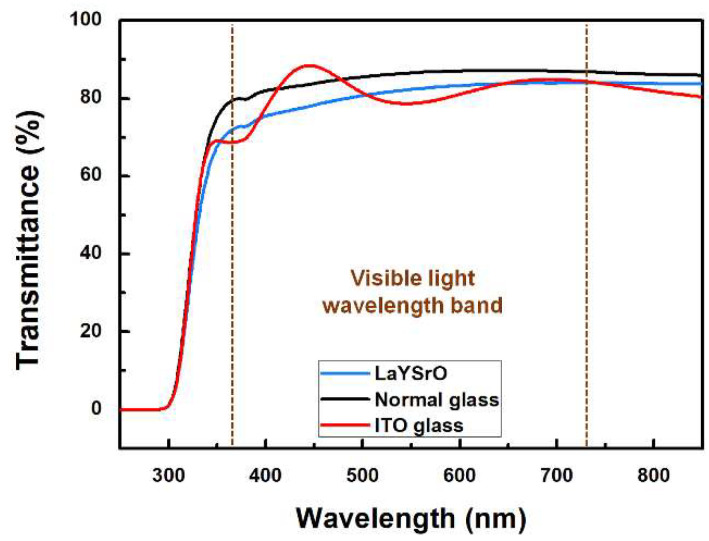
UV transmittance spectra of the brush-coated LaYSrO film cured at 270 °C. Normal and ITO-coated glasses were also measured for comparison. The graphs were obtained in the wavelength range of 250–850 nm, and the band of visible light wavelengths is denoted by the brown dotted lines.

The optical transparency of the brush-coated LaYSrO film cured at 270 °C was investigated in the wavelength range of 250–850 nm, as denoted in Figure 8. The normal and indium-tin-oxide (ITO)-coated glass substrates were also observed for comparison. The wavelength band of the visible light region (indicated by the brown dotted lines in the figure) was considered to be 380–740 nm. In this region, the transmittance curve was an almost straight line without shaking, indicating the uniformity and stability of the film. The average transmittance of the film in this region was calculated to be 81.3%, which was measured as 85.5% and 82.3% for the normal and ITO-coated glasses, respectively. Compared to the plain glass, the light loss occurred on the brush-coated LaYSrO layer because of the directional nano/microstructure on that surface. The quasi-periodic nanostructure can change the optical properties of the film by absorbing and scattering the light [43]. However, the reduction was only 4.3%, and there was no significant transmittance difference compared to the commercially used ITO glass. From the optical transparency analysis, the brush-coated LaYSrO film was found to be stable and suitable as an alignment layer for the LC systems.

**Figure 9:**
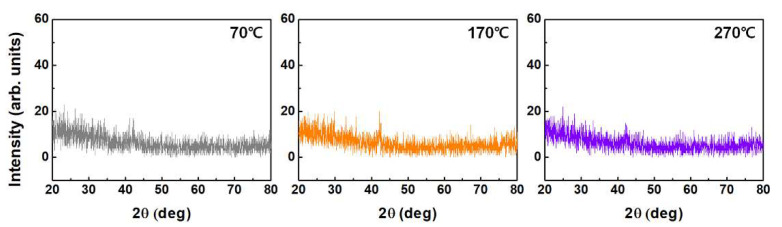
XRD graphs of the brush-coated LaYSrO films cured at 70, 170, and 270 ℃.

To investigate the crystalline characteristic of the brush-coated LaYSrO films according to the film curing temperature, XRD analysis was performed, and the results are presented in Figure 9. The graphs show that there are no major peaks at all curing temperatures, meaning that the brush-coated LaYSrO films have amorphous characteristic. Brush-coating is a solution-based process, and the solution-processed oxide films generally show amorphous characteristic below a curing temperature of 500 °C [44]. Despite the amorphous structure, the LCs were uniformly and homogeneously aligned in the brush-coated LaYSrO layer cured at 270 °C, unlike the layer cured at 70 °C, based on the POM and pretilt angle analyses. From the XRD analysis, it was demonstrated that the amorphous characteristic of the brush-coated LaYSrO films did not affect the uniformity and homogeneous alignment of the LC molecules on the surface.

**Figure 10:**
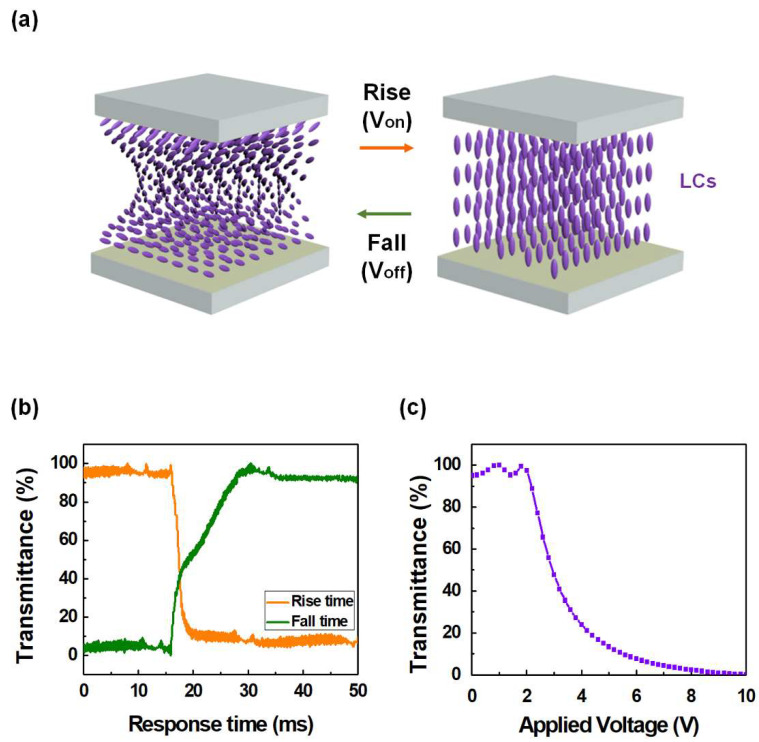
(**a**) Illustration of the operating process of TN-LC cells when a voltage is applied or turned off. EO characteristics of the TN-LC cells fabricated from a brush-coated LaYSrO layer cured at 270 °C: (**b**) response time and (**c**) threshold voltage.

To verify the applicability of the brush-coated LaYSrO layer cured at 270 °C in the TN-LC system, the EO characteristics of fabricated TN-LC cells based on these films were observed. The response time corresponds to the sum of the rise time (transition time of LCs from horizontal to vertical states when a voltage is applied) and fall time (transition time of LCs from vertical to horizontal states when the voltage is turned off), and the operation of the TN-LC cell is as illustrated in Figure 10a. The rise, fall, and total response times were measured and calculated as 3.42, 10.88, and 14.3 ms, respectively, as shown in Figure 10b. This result exhibits the favorable switching characteristic of the LaYSrO layer compared to the conventionally used rubbed polyimide layer [45]; this is attributed to the high dielectric property of lanthanum, yttrium, and strontium oxide, which are the main components of the LaYSrO layer [46,47,48]. The threshold voltage (related to 90% transmittance in the V-T graph) was calculated as 2.17 V, as shown in Figure 10c. This indicates the comparable operating characteristic of the LaYSrO layer with that of the conventional PI layer [19]. From the results, suitable EO performance of the brush-coated LaYSrO layer was demonstrated for application to TN-LC system.

## 4. Conclusions

Herein, we present a convenient and cost-effective orientation method for LC systems. The brush-coating process deposits a LaYSrO film as an alignment layer while simultaneously performing alignment layer treatment to induce uniform LC alignment. The LaYSrO film formation was confirmed by XPS analysis. The three-dimensional morphologies of the LaYSrO films made by spin-coating and brush-coating at various curing temperatures were investigated by AFM. While the spin-coated films denoted isotropic surface and the low-temperature-cured brush-coated films showed no directionality, the brush-coated film cured at 270 °C represented an anisotropic surface with directionality. This directional nano/microstructure was formed by shear stresses through the brush-hair movements during brush-coating. This anisotropic surface induced the uniform LC alignment, and it showed that the brush-coating had successfully performed the alignment layer treatment. The uniform and homogeneous LC alignment state was confirmed by POM and pretilt angle analyses. The brush-coated LaYSrO film showed fine optical transmittance curves with an amorphous property, and the LaYSrO layer-based LC cell showed a suitable thermal budget for LC systems. Appropriate and stable EO performances of the LaYSrO layer were also confirmed in an TN-LC system with a fast-switching characteristic. In this respect, the brush-coating process is notable and can be adopted for LC alignment through a one-step integrated film deposition and alignment treatment process.

## Data Availability

The data presented in this study are available upon request from the corresponding author.
